# Outcome measures in long COVID clinical trials: a review

**DOI:** 10.3389/fpubh.2026.1972605

**Published:** 2026-10-19

**Authors:** Femke C. C. van Rhijn-Brouwer, Chloé de Canson, Joanneke E. L. VanDerNagel, Xandra Westerhuis, Susanne Y. Young, Sander M. J. van Kuijk, Laura V. de Vries, Jon-Ruben van Rhijn, Merel Hellemons

**Affiliations:** 1 Department of Nephrology and Hypertension, University Medical Center Utrecht, Utrecht, Netherlands; 2 Department of Theoretical Philosophy, University of Groningen, Groningen, Netherlands; 3 Department of Human Media Interaction, University of Twente, Enschede, Netherlands; 4 Research Physician, Amsterdam, Netherlands; 5 Department of Primary and Community Care, Radboud University Medical Center, Nijmegen, Netherlands; 6 Department of Clinical Epidemiology and Medical Technology Assessment, Maastricht University Medical Center, Maastricht, Netherlands; 7 Independent Researcher, Amsterdam, Netherlands; 8 Institute for Life Sciences and Chemistry, HU University of Applied Sciences, Utrecht, Netherlands; 9 Department of Respiratory Medicine, Erasmus Medical Center, Rotterdam, Netherlands

**Keywords:** clinical trial design, infection-associated chronic condition, long COVID, myalgic encephalomyelitis (ME), patient reported outcome measure (PROM)

## Abstract

**Background:**

Long COVID is a multisystem disease that affects approximately 10% of people after a SARS-CoV-2 infection. In the absence of curative treatment, high-quality clinical treatment trials are urgently needed for long COVID. There is significant overlap between long COVID and other infection-associated chronic conditions, in particular myalgic encephalomyelitis. As such, trial design for long COVID can benefit from prior experience in this field. In this review we explore outcome measures in long COVID and myalgic encephalomyelitis clinical trials to survey current practices, and provide recommendations for future clinical trials. PubMed and Clinicaltrials.gov were searched for clinical trials on long COVID and myalgic encephalomyelitis that featured a biomedical intervention. 37 myalgic encephalomyelitis and 68 long COVID trials were identified that used 397 outcome measures in total. We then critically assessed the relevance of these outcome measures for patients.

**Conclusion:**

Long COVID trial design could be improved by incorporating outcomes that address important symptom clusters such as post-exertional malaise, dysautonomia and mast cell activation. Clinical assessments, physical tests and biomarkers can supplement patient reported outcome measure data, if safety, feasibility and applicability are carefully considered. Lastly, safety of the intervention is rarely adequately evaluated. We recommend a concerted effort to include patient input at all steps of clinical trial design, execution and evaluation.

## Introduction

Long COVID (LC) is a multisystem disease that develops in approximately 10% of people after SARS-CoV-2 infection (1–3). The majority of people living with long COVID (pwLC) who have LC 6 months after an infection do not recover by either 24 months (4) or 36 months (5). The quality of life in long-term ill people with long COVID is worse than that of advanced cancer patients, with functional impairment comparable to Parkinson’s patients (6). In addition, LC has a major societal and economic impact; over 50% of patients are unable to return to work or fulfill caregiver roles (7, 8). The economic cost of LC is estimated around 1 trillion US dollars yearly, or 1% of the world economy (9).

Although exact LC pathogenesis is unknown, current evidence suggests contribution of viral persistence, immune dysregulation, endothelial dysfunction, and mitochondrial dysfunction (10). The symptom profile of some prominent LC phenotypes closely resembles or even overlaps that of other infection-associated chronic conditions (IACCs) (11). Depending on the cohort, at least half of people with LC meet diagnostic criteria for myalgic encephalomyelitis (ME, also referred to as ME/CFS) (12). LC that meets ME criteria can be considered among the most severe LC phenotypes. The cardinal symptom of ME, and LC with an ME phenotype, is post exertional malaise (PEM) (13). PEM is the new onset or worsening of symptoms after mental, physical or emotional exertion that would not have caused a problem before the illness. PEM may be delayed by 12–48 h after exertion and can last hours to months. It may even result in a chronic deterioration of functional capacity (13). Other ME symptom clusters that overlap with LC are neurocognitive impairments, dysautonomia, mast cell activation syndrome (MCAS) related symptoms, and gastro-intestinal symptoms (14).

To date, there is no curative treatment for LC. A comprehensive overview (15) reported 39 clinical trials underway by May 2024. Given the discovery of promising pathophysiological targets, more interventional trials are expected in the near future.

An important challenge for interventional trials is measuring efficacy. There is a wide range of outcome measurements, such as patient reported outcome measures (PROMs), physical tests, biomarkers and safety data/adverse events. Currently, there is a lack of consensus on which outcome measures to use. While an attempt has been made to establish a core outcome set for LC (16), there is no consensus on how to measure these constructs. More recently, several key opinion leaders and patient-researchers have called for critically rethinking outcomes in LC (17, 18), for example through increased focus on equity (including illness severity) and involvement of patient-researchers (9, 15).

Here, we provide an overview of PROMs, non-PROM outcome measurements, and safety assessments currently used in LC clinical trials, building on the lessons learned from ME clinical trial design. We identify possible knowledge gaps regarding outcome selection in LC clinical trials, and suggest improvements for future research design.

## Approach

Two authors searched PubMed and Clinicaltrials.gov for biomedical trials and compiled lists of outcome measures used in biomedical intervention trials for LC (Supplementary Table 1) and ME (Supplementary Table 2). Reference lists of relevant systematic reviews were hand searched. Only studies that were designed as a biomedical intervention trial from study onset were included, excluding studies that assessed psychological, behavioral, or exercise interventions, as well as studies assessing dietary supplements. The outcome measures were grouped as either PROM, clinical assessments, physical tests, or biomarkers. Clinical assessments are defined as measurements in which the patient is not physically measured (e.g., observer ratings, cognitive testing). Physical tests are measures that include aspects of physical function or report on physical task execution (for example heart rate, lung capacity, but also treadmill exercise tests or cardiopulmonary exercise testing (CPET)). See Supplementary Table 3 for an extended list of all constructs. The construct defined by the original authors of the PROMs was used to inform subgrouping of PROMs into different categories. In LC trials, we identified 261 different outcome measures of which 126 PROMs, 41 clinical assessments, 34 physical tests, and 60 biomarkers (Figure 1A). In ME trials, we identified 175 different outcome measures, of which 92 PROMs, 23 clinical assessments, 25 physical tests, and 35 biomarkers (Figure 1B). Tables and figures were made using Excel version 2,409 (Build 18025.20104).

**Figure 1:**
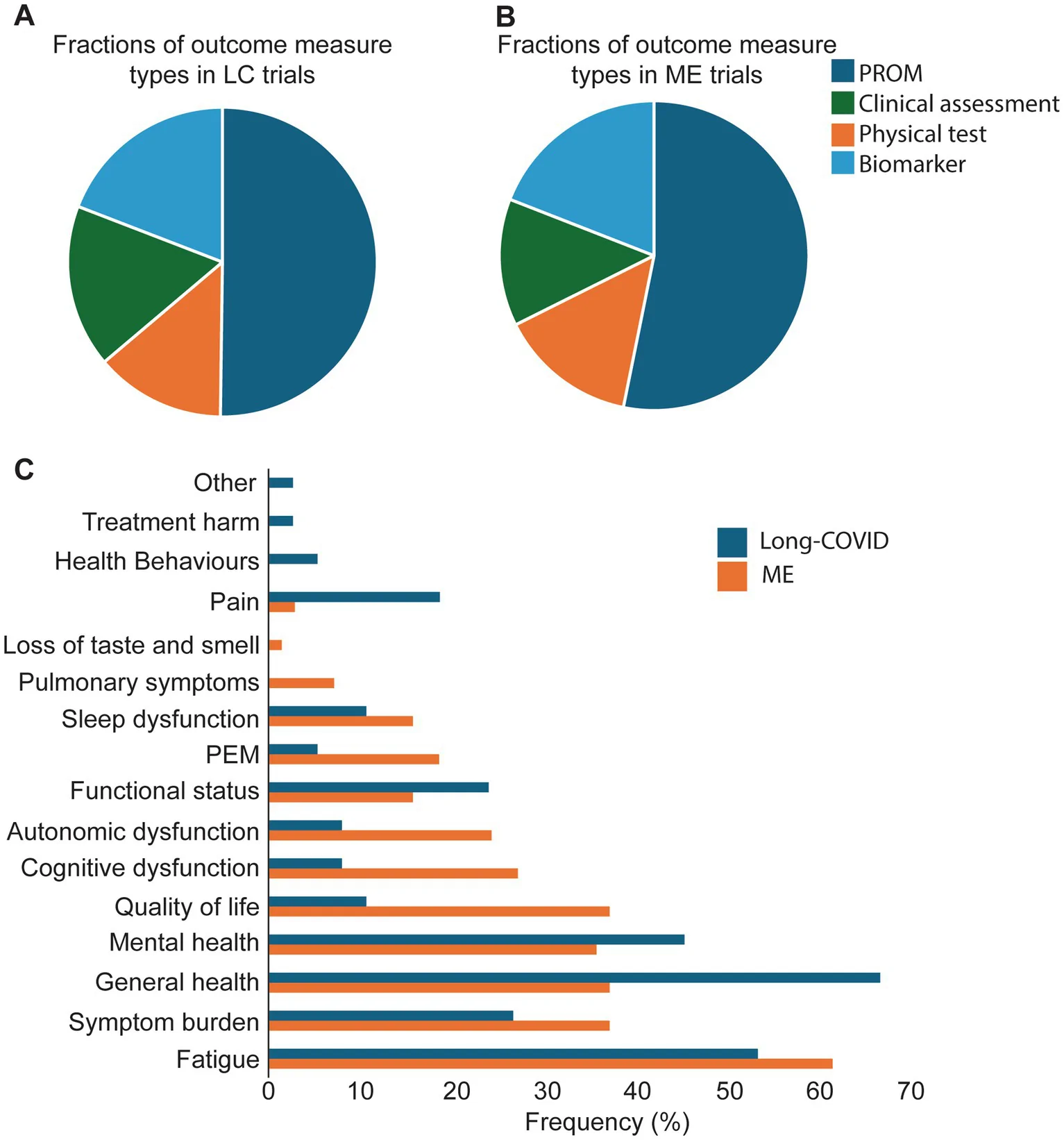
**(A, B)** Outcome measure types used in LC research and ME research, divided into 4 distinct categories. **(C)** Categorization of PROMs into 15 categories (+1 other) following the constructs that they measure according to the PROM developers.

## PROMs

Table 1 provides an overview of different PROM categories used in LC clinical trials. Ten trials used newly developed LC specific PROMs, which mostly consist of symptom inventories, and one assessment of functional capacity. 22 PROMs used in LC trials have been used previously in ME trials. Almost all clinical trials we investigated used PROMs (Tables 2, 3). The PROMs used in LC clinical trials are predominantly pre-existing PROMs, that assess a variety of categories, predominantly fatigue, general health and mental health (Figure 1C).

**Table 1:** Different categories of PROMs used in LC trials.

Author	Year	Fatigue	Symptom burden	General health	Mental health	Quality of life	Cognitive dysfunction	Autonomic dysfunction	Functional status	PEM	Sleep dysfunction	Pulmonary symptoms	Loss of taste and smell	Pain	Health behaviors	Treatment harm	Other
Charfeddine	2022																
Efrati	2022			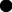	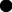	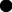					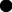						
Arjun	2022	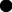	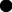														
Finnigan	2023	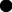															
Momtaz-manesh	2023				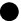												
Geng	2023		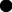	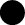													
Achleitner	2023	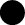															
Stein	2023	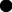							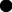								
Ruhe	2023	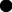		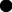	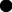	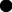											
Prunonosa	2023	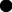	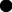		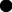	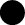	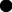		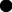								
Redel	2024	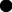			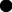		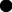										
Krumholz	2024		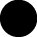	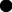	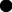	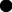											
Isman	2024	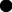		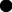													
Brodin	2024	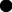				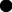	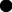	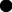		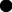							
Argenx (Company)	2024	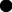	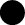				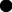	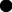									
Posada	2024	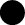	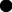														
Thanh Cheng	2024	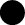	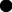		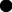						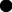		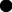	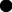			
Strayer	2024	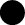	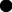	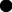	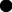		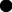				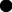						
Leppert	2024	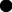			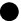	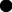	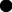										
Houle	2024	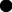	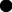	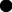		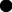				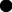							
Putrino	2024	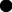		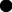		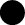		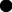		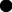		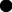					
Nacul	2024	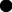	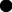		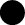	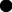					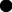						
Walker	2024		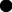														
Saunders	2024																
Reis	2024	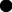															
Banerjee	2024	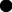			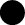	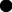			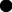			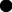					
Kjellberg	2024			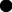		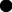											
Pruess	2024	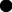	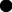	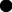	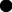			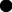		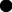							
Tengelmann	2024	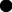	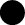		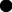	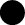	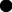		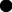								
Argenx	2024	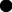	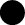				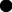	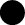									
Jiangsu Pacific Meinouke Bio Pharmaceutical Co. Ltd.	2024																
Guttuso	2024	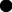		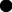	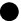	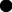	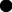				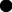						
McIntyre	2024	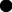		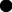	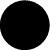	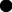	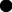		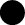								
De Quervain	2024						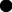										
Anft	2024	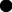	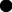					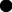	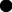		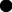						
Peluso	2025			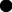				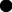									
Henrich	2025		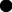	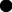													
Zimmerman	2025																
Nath	2025	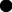	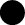	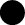													
Mescheder	2025	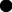															
Schamess	2025				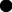		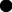										
Audeberg	2025	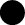			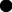	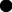	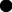										
Bellman-Strobl	2025	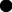	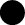	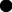	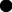			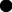		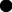							
Putrino	2026					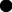											
Zimmerman	2026		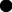	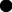				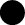									
Yonker	2026		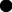														
Putrino	2026					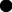											
Zimmerman	2026			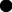				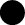									
Siew Chien	2027					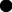											
Wes Ely	2029									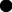							
Putrino	2025	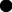		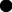	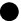	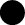	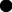	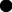		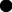	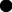						
Evans	2025	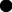		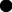	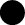	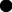						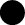					
Weinmann-Menke	2025	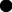	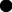			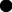		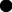	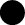	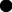							
Fujitani	2025	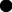			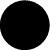	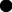											
Burgeois	2028			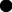	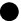			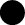			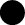	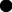					
Klimas	2026	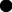	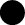	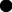	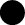			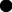		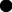	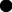	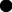		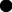			
Barouch	2026	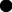				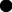											
Bateman	2024	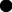															
Fraser	2026		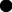	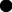	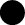												
Nieuwkerk	2028	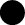		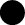	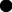		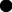	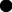		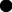							
Wiersinga	2028	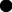		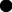			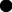	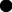									
Van de wijgert	2027	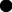		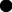			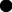	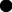		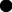							
Shibao	2029																
Scripps Research	2026	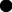				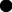			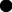	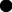							
HealthBio Inc.	2026	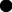	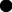	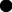			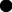	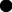									
Paterson	2029					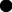											
Markham (biovie Inc)	2026	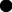					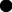			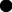	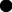						
Lourdes Mateu Pruñonosa,	2026	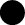		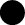	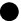	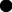			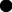		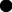						
Sung-Han Kim	2026		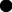														

Additional details can be found in Supplementary Table 1. Dot size corresponds to the number of PROMs in this category used within the trial. Legend: Number of PROMs in this category 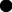 = 1 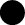 = 2 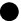 = 3 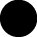 = 4 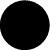 = 5.

**Table 2:** Different types of outcome measures used in LC clinical trials, sorted by trial size.

Author	Year	PROM	Clinical measurement	Physical tests	Biomarkers	*N*
Walker	2024	•	•			5
De Quervain	2024	•	•	•		8
Ruhe	2023	•	•		•	10
Anft	2024	•	•	•	•	12
Yonker	2026	•	•		•	16
Stein	2023	•			•	20
Burgeois	2028	•	•	•	•	21
Prunonosa	2023	•	•		•	25
Achleitner	2023	•			•	27
Peluso	2025	•	•			30
Isman	2024	•				36
Putrino	2024	•			•	40
Henrich	2025	•	•	•		40
Kjellberg	2024	•		•	•	40
Finnigan	2023	•		•	•	41
Momtaz-manesh	2023	•	•			50
Shibao	2029			•	•	50
Guttuso	2024	•				52
Argenx (Company)	2024	•	•	•	•	53
Bateman	2024	•				59
Lourdes Mateu Pruñonosa,	2026	•	•	•	•	60
Schamess	2025	•	•			60
Arjun	2022	•				64
Pruess	2024	•	•	•	•	66
Wiersinga	2028	•	•	•	•	70
Redel	2024	•		•		72
Thanh Cheng	2024	•		•	•	80
Strayer	2024	•	•	•	•	80
Nath	2025	•	•			80
Putrino	2025	•				80
Weinmann-Menke	2025	•	•	•	•	80
Saunders	2024	•		•		83
Putrino	2026	•				90
Putrino	2026	•				90
Zimmerman	2026	•	•	•		90
Barouch	2026	•	•			90
Efrati	2022	•	•	•	•	91
Krumholz	2024	•				100
Klimas	2026	•	•	•		100
Bellman-Strobl	2025	•	•	•	•	104
Fujitani	2025	•	•			110
Posada	2024	•	•			112
Mescheder	2025	•			•	119
Zimmerman	2026	•	•	•		119
Jiangsu Pacific Meinouke	2024		•			121
Evans	2025	•	•	•	•	152
Geng	2023	•	•	•		155
Nacul	2024	•	•	•	•	160
Nieuwkerk	2028	•		•	•	160
Paterson	2029	•	•			182
McIntyre	2024	•	•			200
Leppert	2024	•	•			203
Markham (biovie Inc)	2026	•	•			208
Audeberg	2025	•	•			209
HealthBio Inc	2026	•		•	•	252
Sung-Han Kim	2026	•	•		•	264
Houle	2024	•	•			272
Charfeddine	2022		•		•	290
Tengelmann	2024	•	•	•	•	340
Fraser	2026	•	•	•	•	348
Brodin	2024	•	•	•	•	399
Siew Chien	2027	•	•		•	448
Wes Ely	2029	•	•	•	•	550
Scripps Research	2026	•				1,000
van de Wijgert	2027	•	•			1,250
Reis	2024	•				1,500
Banerjee	2024	•			•	4,520
Zimmerman	2025					N/A

A dot denotes this outcome measure was used in this trial.

**Table 3:** Different types of outcome measures used in ME clinical trials, sorted by trial size.

Author	Year	PROM	Clinical assessment	Physical tests	Biomarkers	*N*
Forsythe	1999	•			•	6
Scheibenbogen	2018	•	•		•	10
Mella	2025	•	•	•	•	10
Peterson	1998	•	•	•	•	20
Peterson	1990	•			•	30
Montoya	2013	•				30
Ishizuka	2027	•	•	•	•	30
Cleare	1999	•				32
Rekeland	2020	•	•	•		40
Young	2024	•	•			44
Joseph	2022			•		45
Lloyd	1990	•	•			49
Roerink	2017	•			•	50
Scheibenbogen	2027	•	•	•	•	50
Blockmans	2006	•				60
Arnold	2015	•	•			60
Nilsson	2018	•	•			62
Pressler	2024	•	•	•	•	66
McDermott	2006	•				67
Rowe	1997	•	•	•	•	70
McKenzie	1998	•			•	70
Blockmans	2003	•				80
Skjevling	2024	•		•	•	80
Hickie	2000	•	•		•	90
Strayer	1994	•	•	•	•	92
Vercoulen	1996	•	•	•		96
Zachrisson	2002	•	•			98
Rowe	2001	•				100
Gottschalk	2025	•			•	100
Sulheim	2014	•		•	•	120
Montoya	2018	•	•			134
Castro-Marrero	2021	•				144
Fluge	2019	•		•		151
Systrom	2026	•	•	•		160
Strayer	2012	•	•	•		234
Blacker	2004	•	•			434

A dot denotes this outcome measure was used in this trial.

## LC specific PROMs

There are several published and validated LC specific PROMs: the Symptom Burden Questionnaire for Long COVID (SBQ-LC) (19), the Munich Berlin Symptom Questionnaire (MBSQ) (20), the Symptom Tool & Impact Tool (ST&IT (21), the Covid-19 Yorkshire Rehabilitation Scale (C19-YRS) (22), and the Post COVID-19 functional status scale (PCFS) (23). These are either symptom inventories (SBQ-LC, MBSQ, C19-YRS, ST&IT) or result in a functional limitation classification (PCFS, scored 0–4).

A review of the ST&IT, SBQ-LC, C19-YRS and the PCFS concluded that only the PCFS satisfied their quality assessment, passing face, content, criterion and construct validity analyses. However, it lacks resolution to measure treatment response, limiting its applicability in clinical trial research (24). For instance, the PCFS would not capture improvement as large as going from fully bedbound to mostly housebound, and the MBSQ was not designed for use in clinical trials and only covers the ME phenotype of LC (20).

Symptom inventories are often limited in their ability to capture symptom clusters such as PEM, neurocognitive impairments, dysautonomia, mast cell activation syndrome (MCAS) related symptoms, and gastro-intestinal symptoms. Another complication in the development of specific symptom inventories is that symptom clusters often involve various organ systems: a person with dysautonomia may have cognitive dysfunction, cardiovascular symptoms and gastrointestinal symptoms. This multidimensionality complicates interpretation of symptom inventories as an outcome measure. For example, the recent Stanford LC Nirmatrelvir/Ritonavir trial (25) (STOP-PASC, NCT05576662, Supplementary Table 1) used a composite outcome based on 6 “core symptoms” of LC that omits highly prevalent symptom clusters such as PEM or dysautonomia.

## PROMs commonly used in ME clinical trials

Table 4 provides an overview of different PROM categories used in ME clinical trials we investigated. LC research often uses PROMs that were previously used in ME clinical trials. Unfortunately, several of the routinely used PROMs in this field have major drawbacks. Below, the most prevalent issues with several of these PROMs are discussed, which include reliability issues, biases based on disproven disease origins, low content validity, lack of patient involvement and ceiling/floor effects.

**Table 4:** Different categories of PROMs used in ME trials.

Author	Year	Fatigue	Symptom burden	General health	Mental health	Quality of life	Cognitive dysfunction	Autonomic dysfunction	Functional status	PEM	Sleep dysfunction	Pulmonary symptoms	Loss of taste and smell	Pain	Health behaviors	Treatment harm	Other
Peterson	1990		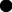	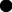													
Lloyd	1990				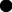	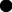											
Strayer	1994			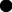	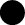												
Vercoulen	1996	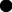			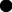				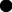						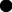		
Rowe	1997			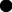	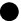	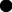											
McKenzie	1998			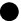	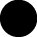												
Peterson	1998			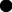	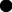												
Forsythe	1999		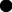														
Cleare	1999	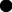	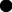	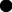					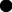							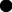	
Hickie	2000			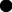	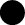												
Rowe	2001			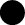	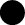		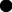		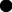								
Zachrisson	2002			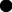	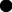									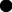			
Blockmans	2003	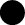		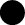	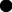												
Blacker	2004	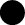		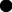							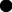						
McDermott	2006	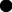	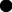		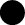	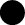											
Blockmans	2006	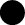		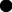	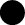												
The	2010	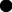		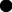											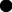		
Strayer	2012			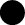					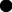								
Montoya	2013	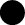		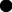	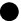						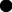						
Sulheim	2014		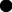			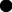		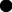						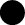			
Arnold	2015	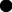	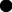		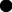									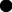			
Roerink	2017	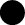		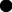										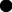			
Nilsson	2018	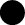			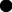									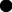			
Montoya	2018	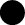					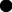										
Scheibenbogen	2018	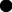	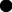														
Fluge	2019	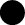		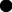	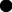				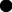								
Rekeland	2020	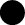		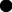					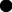								
Castro-Marrero	2021	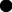		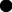							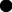						
Joseph	2022																
Skjevling	2024	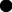	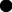	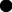	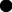												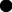
Pressler	2024	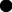	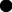	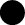					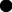	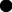							
Young	2024	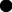	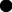				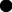										
Gottschalk	2025			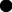													
Systrom	2026								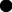	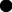				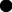			
Mella	2025			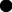					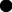								
Ishizuka	2027			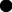				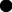			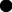			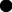			
Scheibenbogen	2027	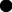		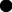				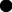									

Additional details can be found in Supplementary Table 2. Number of PROMs in this category 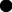 = 1 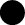 = 2 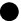 = 3 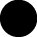 = 4 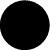 = 5.

Systematic reviews (26, 27), show that several widely used PROMs such as the Beck Depression Inventory (BDI) or the Fatigue Severity Scale (FSS) provide no or limited published evidence for reliability (test–retest or internal consistency), validity (content or construct validity), or responsiveness in pwME.

Moreover, many PROMs used in ME research were not based on qualitative research, but on preconceptions that ME and other IACCs have a psychosomatic origin. These PROMs, for example the Checklist Individual strength (CIS) (28) and the Chalder Fatigue Scale (CFS) (29) perpetuate outdated notions that ME is the result of false illness beliefs adopted by patients in order to benefit from a sick role (30–32). This theory has since been disproven (12, 33), nevertheless, these PROMs are still widely used (Supplementary Table 2).

Indeed, the CFS is the only ME specific PROM that has been evaluated for content validity by cognitive interviewing (the current standard of content validity evaluation) and it performed very poorly. Interviewed pwME stated that the instruction text asked a very difficult task, namely to rate according to their life before illness, and that 5 of the 11 items lack clarity, which impaired their ability to rate themselves (34).

It should also be noted that in general there was a lack of patient-involvement in development and evaluation of ME-specific instruments. This has led to a gap between what PROMs intend to measure and what is actually measured, as was recognized recently (35). Researchers could therefore benefit from a keen awareness of these controversies, enabling informed decision making on using PROMs that presume a psychosomatic origin of ME.

Other important limitations of ME specific PROMs have been described. Ceiling and/or floor effects are prevalent in PROMs used in ME and LC trials (34, 36). This has been reported for the CFS (37), CIS and FSS (38), and the SF-36 (39). Using these PROMs may conceal treatment effectiveness, as any change that does not lead to a score noticeably below ceiling or above floor will be missed. Another concern is that for persons already at ceiling, further deterioration cannot be recorded. Ceiling effects therefore particularly compromise the assessment of persons with severe disease; a group that is most in need of treatment and intervention trials. This is in particular relevant regarding assessment of treatment safety.

Lastly, a more general concern with PROMs used in ME research is that people with PEM are often medically advised to pace, that is, adjusting their activity levels to stay within their energy envelope to avoid PEM (40) and prevent possible disease progression. This limits the usability of PROMs that assess frequency and severity of symptoms, as the (relative) absence of symptoms due to successful pacing may lead to an inadequate assessment of how an individual is affected (41). In addition, access to home health care and mobility aids may affect scores as meeting support needs enables more successful pacing. Intriguingly, a correlation between pacing effectiveness and increased scoring on the SF-36 was recently shown (42), as such, symptom burden and functionality are interdependent.

## Constructs frequently used in PROMs

In order to comprehensively capture the effects of interventions in clinical trials, PROMs should be both specific to a disease, and be able to detect small differences between individuals. PROMs often investigate specific constructs (i.e., phenotypes or concepts associated with a disease). We assessed the most frequently used constructs in PROMs that were employed in either LC and/or ME clinical trials.

## Fatigue

Our analysis revealed that more than 54% of PROMs that are used in LC or ME clinical trials predominantly assess the construct fatigue. Although fatigue is often seen as the key symptom in LC and ME, it does not adequately describe the lived experience of pwLC: what is called fatigue in ME and LC, is not what is commonly known as fatigue (43). This might explain why cognitive interviewing showed that pwME felt unable to answer the CFS fatigue items. Moreover, modern ME criteria no longer require the presence of fatigue for a diagnosis (13).

In addition, most fatigue PROMs are binary instruments leading to a classification of “clinically relevant fatigue” above a certain arbitrary threshold. They are not designed to detect a treatment effect, even though for some PROMs, Minimal Important Differences (MIDs) have been established (44).

## Post-exertional malaise

PEM can present highly variably. Some report changes to multiple symptoms, such as tolerance to light and sound, nausea, a flu-like syndrome, or muscle weakness, whereas others report debilitating symptoms in only one system, for example increased severity of headaches. PEM is highly disabling, and there is currently no gold standard for the diagnosis of PEM. The most widely accepted objective measure of PEM (two-day cardiopulmonary exercise test (CPET)), risks severe iatrogenic damage as we discuss below. This all highlights the need for adequate PROMs that capture the full scope of PEM.

PROMs that specifically assess PEM are only used in 18% of LC and 5% of ME trials, and these use variants of the DePaul symptom Questionnaire Post Exertional Malaise (DSQ-PEM). The DSQ-PEM 5 and DSQ-PEM 10 are commonly used to screen for PEM in LC clinical trials and biomedical research. However, the DSQ-PEM does not consider symptom delay (which is frequent in PEM) and patients may feel unaffected during or directly after exercise. The DSQ-PEM also mostly focuses on fatigue whereas patients report many other symptoms during a PEM episode. Lastly, results from the DSQ-PEM are affected by pacing (as described above). New versions of the DSQ-PEM have been developed to address these concerns (42).

The lack of proper assessments for PEM is a core issue for clinical research into LC and other PAIS/IACC, and calls for development of PROMs that adequately assess PEM have been made in the last decade (15, 27, 36, 45).

## Dysautonomia

Common forms of dysautonomia in pwLC and pwME are postural orthostatic tachycardia syndrome (POTS) and orthostatic intolerance (OI). Patients typically report orthostatic worsening of symptoms, head/neck discomfort (so called “coat hanger pain”), cognitive impairment and/or gastrointestinal complaints (46). Three PROMs were used to assess dysautonomia in pwLC: the COMPASS-31, the OHD/OIQ, and the Malmö POTS inventory. The COMPASS-31 is used in 21% of trials, the OHQ/OIQ is used in 5% of trials, and the Malmö POTS inventory in 2% of trials. While the COMPASS-31 in particular may be useful to detect general features of dysautonomia, it has insufficient face and construct validity to diagnose POTS/OI as it focuses predominantly on presyncope symptoms, which are not present in 45% of pwLC with an abnormal NASA lean test (46). The OHQ/OIQ and the Malmö POTS inventory do include non-presyncope symptoms such as head/neck discomfort and weakness. In addition, the OHQ/OIQ also includes functional consequences of orthostatic intolerance, and the Malmö POTS inventory also includes fatigue and gastro-intestinal problems that might be part of other symptom clusters. Self-reported hours of upright activity might reflect the severity of POTS/OI, and have been recommended by thought leaders (47). However, to date this has not been implemented in LC or ME trials (48).

## Mast cell activation syndrome (MCAS)

In MCAS the inappropriate secretion of mast cell mediators leads to symptoms in several organ systems (49). MCAS can trigger or worsen PEM and dysautonomia. A subset of pwLC reports clear mast cell mediated symptoms such as pruritus and flushing after histamine exposure or histamine releasing medication (49, 50). Additionally, many pwLC respond to MCAS treatments, even if they do not display classical MCAS signs and symptoms (51).

MCAS-related symptoms are currently not addressed in clinical trials even though there is at least one validated PROM for MCAS (52, 53).

## Mental health outcomes

Mental health outcomes are the second most prevalent outcome measure used in LC intervention studies. The most widely used PROMs for the assessment of mental health in LC are the PHQ-9 for depression and the GAD-7 for anxiety. A recent systematic review, however, found insufficient data to assess the content or face validity of these PROMs in LC (54).

Mental health PROMs commonly assess highly prevalent physical symptoms of LC, that are independent from mental status (15). The PHQ-9 for instance explores tiredness, appetite and sleep problems and attributes these to depression and anxiety. Likewise, symptoms scored by the GAD-7, such as “trouble relaxing,” or “feeling afraid, as if something awful might happen”, also occur in OI or MCAS and may thus not necessarily indicate an anxiety disorder. Mental health PROMs also do not consider the impact of loss of quality of life due to disease severity. Higher rates of depression, anxiety, and suicidality are associated with greater symptom severity and limitations in physical capacity (55). Importantly, mental health PROMs are designed for screening and not for diagnosing conditions (56, 57) or to replace face to face interviews, as they may overestimate the prevalence of, e.g., depression.

## Measuring severity and functional capacity

LC related disability can range from isolated anosmia to being bedbound and unable to tolerate any stimuli (58). 16% of trials use PROMs that inquire about functional status. However, specific inquiry on severity is sparse, happening only in 8% of trials, and these trials often do not use previously established rating tools. In ME, various scales and rating tools exist to assess severity, such as the self-rated Bell disability scale (59) and the Symptom Severity Impact from the international consensus criteria (ICC) for ME (43). The Bell score provides a framework for patients to describe the impact of their symptoms on daily life. The ICC are based on a large Delphi study and classify patients as Mild (functional capacity reduction of at least, 50%) Moderate: mostly housebound, Severe: mostly bedbound, and Very Severe: fully bedbound. Finer grained but more complex versions have been proposed (43).

Scales that assess ME and LC severity often suffer from a ceiling effect. For instance, the PCFS ranks patients on a scale from 0 (least affected) to 4 (most affected), where anyone who cannot live alone without assistance is classified as a 4 (23). However, those classified as “very severe”, “severe”, and probably a subset of those classified as “moderate” according to ICC, would be classified as a 4 according to these criteria. In addition, a PROM like the cognitive failure questionnaire assesses how often people encounter problems in a set of activities, such as with shopping for groceries, yet does not include an option to score the inability to perform a task.

Currently, the only PROM which has been validated against the ICC severity scale is the FUNCAP (41). This relatively novel PROM was created with extensive patient involvement and measures the functional consequences that various activities will have on the patient. It has been deemed particularly promising by patient-researchers (17), but remains to be validated in LC phenotypes beyond ME.

Return to work, including the number of hours and the quality content of the work, is an important holistic signifier of functional capacity, but rarely reported in intervention studies in pwLC.

## Quality of life

38% of trials use quality of life (QoL) as an outcome measure. This reflects the recent increase in advocacy for health-related QoL as a primary outcome in treatment research (60). In a meta-analysis of PROMs in LC, the EuroQol EQ-5D-5L had the best psychometric properties (54). However, researchers and patients have called for better QoL PROMs (61), since QoL PROMs often poorly reflect patient-identified QoL impacts (62) and patients are often not involved in PROM design (63).

There are several factors that complicate using QoL measures as primary outcomes. Small changes in QoL often cannot be captured (39). Currently available off label treatments are not expected to lead to major health improvements but even an increase in upright hours of activity of 15 min can represent a very valuable result for severely affected patients.

Experience of QoL can also be subject to bias. For instance, it does not take into account the effect of being in a trial. Being in a trial or a cohort where your symptoms are taken seriously is a stark contrast with “regular care” where patients frequently experience gaslighting. Additionally, many QoL measures do not consider the significant challenges of living with an energy limiting illness. Someone may feel quite well but only due to pacing stringently.

## Non-PROM outcome measures

Non-PROM outcome measures were used in 80% of LC and ME trials (Tables 2, 3). 17% of ME trials and 25% of LC trials have a non-PROM primary outcome measure. Below, we discuss the predominant outcome measures in these categories, and their opportunities and drawbacks.

## Clinical assessments

ME and LC trials that use clinical assessments, mostly use neurocognitive tests (48%), or observation-based assessments (29%) (e.g., the Clinician Graded Impression of Change, CGI-C). In contrast to reporting in acute cohorts, no trials capture factors such as return to work, or conventional outcome measures such as survival or major cardiovascular events (64, 65). In 12% of all ME trials neurocognitive tests are used, while 37% of LC trials employ some form of cognitive testing as an outcome measure. Trials employ either general tests for cognitive decline, such as the Mini Mental State Exam (MMSE) or the Montreal Cognitive Assessment (MoCA), or specific task-related tests such as Digit Span Tests (DST). Such assessments are designed to measure cognitive decline, for example related to neurodegeneration. Therefore, they are not suited to screen for neuropsychological symptoms in LC or ME trials, as only severe cognitive dysfunction will be captured by these assessments. Furthermore, they do not take into consideration the sometimes dynamic nature of cognitive dysfunction in pwME and LC, nor do they capture the PEM after such testing.

## Physical tests

Physical tests range from general (such as heart rate, blood pressure) to specialized investigations (for example: MRI, CPET), or assessments of cardio-pulmonary fitness (for example: 6-min walk test (6MWD/6minWT), 30 s sit to stand test). 45% of LC trials and 44% of ME trials include such physical tests. The most commonly used tests investigate dysautonomia (e.g., NASA lean test (NLT)) and exercise capacity (6MWD, treadmill tests). 14% of LC studies that use non-PROM outcome measures use an active stand test.

Although the tilt table test is still considered by some as the golden standard to investigate orthostatic tachycardia, no studies in our investigation used this as an outcome measure for a clinical trial. Currently, there is consensus that orthostatic vital signs are generally sufficient for a diagnosis (66). Tilt table tests should only be considered when orthostatic vital signs investigation (for instance in the NASA lean test) is negative. Other factors to consider in dysautonomia tests are that circadian rhythm and exertion (e.g., travel to the clinic) may affect readout results. It has been shown that changes in cerebral perfusion correlate well with orthostatic symptoms but this measurement has not yet been implemented in clinical trials so far (67).

Tests that involve induced activity such as the 6MWD should be considered with caution, as these often result in PEM. Early LC research built on the assessments used to investigate post-SARS1, such as the 6MWD. However, there are striking differences between the SARS1 population and LC/ME. For example, all SARS1 patients had been hospitalized and most required ICU support – a population that is likely to have a decreased 6MWD. The mostly not hospitalized LC population however, might not demonstrate a diminished cardio-respiratory capacity, but develops PEM afterwards. Thus, the added value of such assessments in LC can be questioned.

Two-day cardiopulmonary testing (CPET) clearly demonstrates PEM (68), as pwPEM will have a markedly reduced performance on day two. However, its use should be carefully balanced against the risk for the patients involved. Currently, only single session CPETs are used in clinical trials (4% of trials). Long term follow-up is rarely reported. In one study involving pwME, most patients had a relapse that lasted at least 2 weeks following 2-day CPET, and in one case disease deterioration lasted for over a year (69).

The hand grip strength test, used in 10% of studies, is a less strenuous test that accurately assesses fatiguability and is an objective measure of disease severity in pwPEM (70). Both reduced general strength and impaired recovery within a 1-h interval have also been described for pwLC (71). For severely ill patients, measuring PEM from regular activities should be considered.

Another option is to characterize daily life activity. Interestingly, early ME trials (72, 73) did this, but these measurements were rarely employed in the past 20 years. In our investigation only 6% of LC trials used these data (Supplementary Table 1). The recent advent of smart devices (smart watches, rings, phones) provides novel opportunities to assess activity (i.e., daily steps), as well as heart rate, sleep patterns or sleep quality.

## Biomarkers

Biomarkers are often based on specific hypotheses related to trialed intervention. These range from serum lactate levels to inflammation markers or fecal metabolites. 49% of ME trials and 49% of LC trials include biomarkers. They can be valuable measurements within the context of a specific intervention.

It is often posited that finding a single unique biomarker to diagnose LC is the solution to the problems described above with PROMs, as such a biomarker would bypass the need for PROMs altogether. However, firstly, biomarkers identified in LC and ME wax and wane over time within an individual (74–76). Moreover, any biomarker or test with potential for diagnostic use needs to be validated against an independent measure of disease severity, and the only way to independently measure disease severity is to employ PROMs. Improving the quality of PROMs used in clinical trials might result in the identification of more accurate biomarkers. In the absence of a comprehensive understanding of underlying pathophysiology, a biomarker could only ever be a proxy, dependent on the previously established PROM outcomes with which it seems to correlate.

## Measuring safety in clinical trials

There are specific safety concerns in trials for LC and ME. Because many people with LC have PEM, and PEM can lead to permanent baseline decline, every trial that requires exertion on the part of the participant can lead to harm. This includes factors such as talking, filling out questionnaires, or traveling to the study location.

Even while more protracted and permanent cases of PEM meet the formal definition for a serious adverse event (SAE), measuring PEM is not yet routinely implemented in safety protocols or termination criteria. Therefore, it is crucial that LC and ME trials measure severity and length of all PEM episodes that might be causally related to the study. To capture more delayed impact, trials should consider to continue to measure PEM for some time after the intervention has ended. In a recent publication (77) that assessed HBOT, 56% of people improved, but 31% did not respond and 13% showed a negative response at the primary outcome measure (SF-36) 3 months after the intervention. 4% of patients dropped out prematurely because of worsening of symptoms. Such data is rarely reported, but essential for informed and shared decision-making.

PwPEM also have a particular susceptibility to adverse reactions from medications (78). The reasons for this are unclear. As with PEM, these reactions sometimes lead to protracted or even permanent deterioration, that may not be recognized as a drug related adverse event (AE). In addition to (serious) AE reporting by skilled personnel, it is important to employ measures for functional capacity into stopping criteria.

## Reinfection

(Re)infection with SARS-CoV-2 or other viruses is a neglected, but highly relevant, event. It can be measured both via PROMs and through biomarker measurement. In 3% of the trials in our investigation current infection status was assessed at trial start, but the effect of recurrent infections was not explored. Viral (re)infection can have a major negative effect on LC disease progression (79) and may as such influence study outcomes. It might also indicate immunosuppressive effects. However, reinfections are not routinely monitored in trials. Active screening and implementation of protective measures during on location visits may reduce confounding by reinfections.

## Discussion

In this review, we provide an overview of outcome measures used in existing LC and ME clinical trials, with the aim to survey current practices and provide recommendations for future clinical trials. LC research widely uses patient-reported outcome measures (PROMs). Researchers in this field should be aware that some of the PROMs frequently used in the past are outdated and less appropriate, as these have an incorrect assumption of a psychosomatic origin. Fortunately, thinking about LC rapidly evolved to considering LC as a chronic multisystem disease with significant overlap with ME in 50% of cases. Core outcome sets and PROMs should reflect this evolution to enable development of effective treatments. Supplementary Table 4 provides an overview with considerations and limitations of outcome measures mentioned in this manuscript.

Here, we show that LC clinical trials often use outcome measures that have poor content or face validity. Also, outcome measure selection often does not reflect prevalent symptoms and symptom clusters as reported by pwLC after 6 months (80). This requires thorough reassessment of core concepts and decisions surrounding clinical trials. Our suggestions for a best practice approach are outlined in Table 5.

**Table 5:** Best practices for selection of relevant outcome measures in clinical trials.

We recommend to	We recommend not to
Use a broad symptom inventory in conjunction with construct specific PROMs	Use PROMs outside of their intended context
Include non-PROM outcome measures such as actimetry, return to work, hours of upright activity and (re)infections, especially in interventional studies	Use fatigue PROMs as (sole) primary outcome measure
Use measurements that differentiate across the illness severity spectrum, including in the (very) severe range	Use mental health scales that include physical symptoms that are part of LC/ME, leading to diagnostic misclassification
Use outcome measures that assess the presence and severity of symptom clusters like PEM, dysautonomia and MCAS	Use measurements with large ceiling and floor effects
Ensure that harms are correctly recorded, including PEM and baseline decline	Use screening instruments for cognitive problems that only capture severe disability
Screen for reinfections and aim to prevent these	
Involve pwIACCs in all aspects of trial design	
Design novel PROMs, specific to the construct that is being investigated	
Record major cardiovascular events and survival	

Recently, several perspectives on improving LC clinical trials have been published (15, 24). One of the most important problems to be solved remains the broad case definition of LC. While this promotes health equity, it also introduces significant heterogeneity in clinical trial populations. Clinical trials often rely on the selection of limited and sometimes even a single cardinal symptom, such as fatigue, as a proxy. This diminishes the patient experience and foregoes the possible interactions between symptom clusters. Each of the core symptom clusters spans many symptoms from different organ systems. This means that symptoms belonging to multiple syndrome clusters may confound the data if a single cardinal symptom is used to construct outcome measures.

Active involvement of patients in the entire trial process, from selecting relevant outcomes in clinical trial design, to the interpretation of data, is essential. Currently used PROMs should be critically assessed and retired when it is found they do not adequately reflect the patient experience or suffer from core design flaws, such as ceiling effects, misattribution (e.g., somatic symptoms are interpreted as proxies of anxiety or depression) or observer bias.

In both PROM and non-PROM outcome measures, standardization should not be an aim in itself. While data comparisons may be of use, this does not outweigh the cost of incorrectly annotating cohorts with unsuitable outcome measures. A pragmatic approach should be taken, and questions added and validated based on iterative research, supported by patients.

Patient-relevant measures such as hours of upright activity and return to work, and clinical measurements such as actimetry can support data from functional capacity PROMs and deserve a more prominent place in clinical trials. A recent systematic review showed that LC is associated with cardiovascular symptoms and conditions, including major cardiovascular events (81). All LC clinical trials should therefore record these.

Biomarkers can be of added value in conjunction with PROMs, but should not be seen as a replacement. Any development of outcome measures should keep patient relevance as a focus (15).

This review only considered outcome measures in clinical trials. LC cohort studies mostly employ the same PROMs as used in biomedical trials. In a large systematic review and meta-analysis of 63 cohort studies on LC the use of QoL PROMs was assessed (82), and it was reported that 15 of these 63 studies included PROMs related in some way to quality of life. Another review on PROMs used in cohort studies showed that unstandardized symptom inventories are frequently used, and validation of PROMs is often lacking (54). As such, it is clear that cohort studies should be subject to the same considerations as the clinical trials surveyed in this paper.

Children are an underrepresented population in LC research and trials. Regulatory issues often complicate their inclusion. However, given that 10–20% of children are affected by LC following infection (83), with the estimate that children will be similarly affected as adults, outcome design should also take into account this group. Validation of new PROMs should include children, and reference ranges for biomarkers and physical tests for children should be established.

Our final suggestion is that outcome measures should be designed to capture the entire disease spectrum from mild to very severe. At least 19% of pwLC are severely limited (84). The more severely affected the participants are, the more likely they are to suffer from PEM as a result of participating in a trial; and severely or very severely affected patients will not be able to participate in trials without accommodations.

## Conclusion

Taken together, we think that it is essential to learn from the past, in order to improve trial rigor. We aim to inspire current and upcoming clinical trials to critically assess their approach, and to review the assumptions and biases that might be present in the PROMs and outcome measures they use.

## Glossary

6MWD: 6-min walk test
BDI: Beck Depression Inventory
C19-YRS: Covid-19 Yorkshire Rehabilitation
ScaleCIS: Checklist Individual strength
CFS: Chalder Fatigue Scale
CPET: cardiopulmonary exercise testing
DSQ-PEM: DePaul symptom Questionnaire Post Exertional Malaise
DST: Digit Span Tests
FSS: Fatigue Severity Scale
IACC: Infection-associated chronic conditions
ICC: International Consensus Criteria
LC: Long COVID
MCAS: Mast Cell Activation Syndrome
ME: Myalgic Encephalomyelitis
MID: Minimal Important Differences
MMSE: Mini Mental State Exam
MoCA: Montreal Cognitive Assessment
MSBQ: Munich Symptom Burden Questionnaire
NLT: NASA lean test
OI: Orthostatic Intolerance
PCFS: Post COVID-19 functional status scale
pwLC: People with Long COVID
PEM: Post Exertional Malaise
POTS: Postural orthostatic Tachycardia Syndrome
PROM: Patient Reported Outcome Measure
SBQ-LC: Symptom Burden Questionnaire for Long COVID
SAE: Serious Adverse Event
ST&IT: the symptom tool & impact tool
QoL: Quality of Life
